# Common theme for drugs effective in overactive bladder treatment: Inhibition of afferent signaling from the bladder

**DOI:** 10.1111/j.1442-2042.2012.03196.x

**Published:** 2012-10-17

**Authors:** Brandy Hood, Karl-Erik Andersson

**Affiliations:** 1 Institute for Regenerative Medicine, Wake Forest University School of MedicineWinston-Salem, North Carolina, USA; 2 Department of Urology, Wake Forest Baptist Medical CenterWinston-Salem, North Carolina, USA

**Keywords:** mirabegron, mucosal signaling, myogenic pathway, onabotulinum toxin A, tadalafil

## Abstract

The overactive bladder syndrome and detrusor overactivity are conditions that can have major effects on quality of life and social functioning. Antimuscarinic drugs are still first-line treatment. These drugs often have good initial response rates, but adverse effects and decreasing efficacy cause long-term compliance problems, and alternatives are needed. The recognition of the functional contribution of the urothelium/suburothelium, the autonomous detrusor muscle activity during bladder filling and the diversity of nerve transmitters involved has sparked interest in both peripheral and central modulation of overactive bladder syndrome/detrusor overactivity pathophysiology. Three drugs recently approved for treatment of overactive bladder syndrome/detrusor overactivity (mirabegron, tadalafil and onabotulinum toxin A), representing different pharmacological mechanisms; that is, β-adrenoceptor agonism, phosphodiesterase type 5 inhibition, and inhibition of nerve release of efferent and afferent transmitters, all seem to have one effect in common: inhibition of the afferent nervous activity generated by the bladder during filling. In the present review, the different mechanisms forming the pharmacological basis for the use of these drugs are discussed.

## Introduction

The OAB, defined either symptomatically as the OAB syndrome or urodynamically as DO, is a bladder filling disorder. To exert normal bladder control, adequate sensory input to the CNS is necessary, and it is well established that changes in sensory mechanisms might give rise to disturbances in bladder function. It is therefore logical that pharmacological control of bladder contraction has focused on how afferent nerve activity is generated peripherally and handled by the CNS. Several factors might contribute to the genesis of OAB, and at least two afferent signaling pathways in the bladder can be identified, the “myogenic” and the “urothelial” pathway.^1^

It is obvious that the mechanisms leading to an increased activity in afferent nerves might be interesting targets for drugs aimed at controlling sensory and motor activity of the bladder. It is now widely accepted that antimuscarinic drugs, which are still first line treatment of OAB/DO, exert effects on afferent signaling important for their clinical efficacy.^2^ Three novel drugs have recently been approved for treatment of OAB/DO: mirabegron, tadalafil and onabotulinum toxin A. They represent different pharmacological mechanisms; that is, β-AR agonism, PDE5 inhibition, and inhibition of nerve release of efferent and afferent transmitters, and why they all are clinically effective has not been definitely established. In the present review, the mechanisms providing the pharmacological basis for the use of these drugs are discussed.

## Bladder afferent signaling

The bladder mucosa is richly innervated with afferent nerves carrying information from the bladder to the CNS, but also the detrusor muscle is supplied with such nerves. The fibers most important in the control of micturition are myelinated Aδ fibers and unmyelinated C-fibers.^1^ Aδ fibers are mechanosensitive, discharging in response to distension of the bladder wall. C-fibers are normally inactive, but might respond to, for example, chemical irritation or stretch of the urothelium. Under pathological circumstances, C-fibers can be recruited to create new sensory pathways.

As mentioned, afferent fibers in the bladder are activated through nervous pathways in the detrusor muscle (“myogenic”) and the mucosa (“urothelial”).^1^ During bladder filling, there is no excitatory parasympathetic outflow from the CNS,^3^ but there is a myogenic contractile activity contributing to bladder tone. This contractile activity is most probably influenced by activity in the sympathetic nerves to the bladder and in local nervous circuits, resulting in what is referred to as spontaneous (autonomous) contractile activity.#b^4^b^5^ The constant afferent output from the bladder generated by this autonomous activity has been termed “afferent noise”.^6^ The urothelium and suburothelial interstitial cells might exert influence over afferent nerves in response to stretch or various chemical mediators,#b^7^b^8^ representing another source of constant afferent input.

**Fig. 1:**
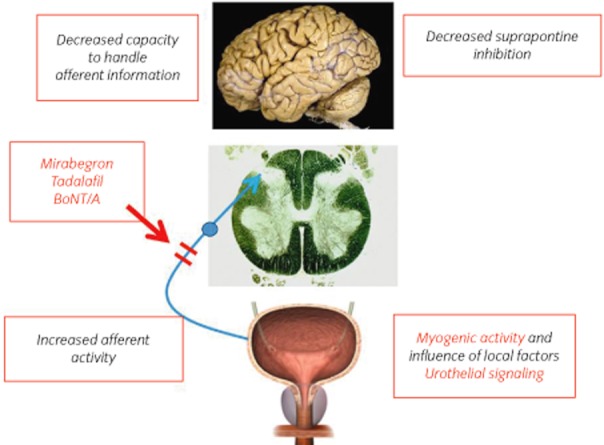
Pathophysiology of the OAB. In the bladder, increased afferent activity generated in the detrusor or mucosa through different mechanisms initiate involuntary detrusor contractions. Such contractions can be initiated independently of changes in afferent activity by changes in the CNS.

There are many theories regarding the pathogenesis of OAB (Fig. 1), but it is likely that abnormally increased signaling through afferent pathways might be involved. Therefore, pharmacological manipulation of the mechanisms generating these signals might be effective in the treatment of OAB. It should be emphasized that the afferent signals generated by the bladder have to be processed in the CNS (Fig. 1). This is an important step in OAB pathophysiology, and a promising but challenging therapeutic target.

## Pharmacological control of bladder afferent nerve activity

### β-AR agonism

In the human detrusor, the β_3_-AR has been found to be the predominant subtype.#b^9^b^10^b^11^ The β_3_-AR, likely along with β_2_-AR, is directly involved in detrusor smooth muscle relaxation.#b^4^b^10^b^11^ How this effect can be translated into the *in vivo* effects of β_3_-AR stimulation seen in numerous preclinical studies and clinical trials – increased bladder capacity without change in micturition pressure or residual volume – has not been clearly established. The normal stimulus for activation of the micturition reflex is distension of the bladder. This initiates activity in “in series”-coupled, low-threshold mechanoreceptive (Aδ) afferents.^12^ If the compliance of the bladder is increased, the response to distension is decreased; and to recruit afferent activity sufficient to initiate micturition, greater filling volumes are needed – thus bladder capacity increases. One of the factors that generates bladder tone is the autonomous contractile, phasic activity of the detrusor smooth muscle that occurs during filling. These contractions are believed to generate afferent input from both Aδ- and C-fibers, which together with the activity initiated by distension, will start the voiding reflex.

The autonomous activity of the detrusor is effectively reduced by β_3_-AR agonists,^13^ and this reduces the afferent output from the bladder. In fact, Aizawa *et al*. showed in rats that the β_3_-AR agonist, CL316243, could inhibit filling-induced activity not only in mechanosensitive Aδ-fibers, but also in C-fiber primary bladder afferents provided that these fibers were stimulated by PG E_2_.^14^ Even if β_3_-AR agonists *in vitro* might be able to relax detrusor muscle contracted by muscarinic receptor stimulation using high concentrations of, for example, ACh or carbachol, they are not very effective. The voiding contraction is caused by a massive release of contractant transmitters (ACh and ATP), and this might be the reason why it is not affected by β_3_-AR agonists. Providing further evidence that β_3_-AR agonists might influence the sensory regulation of micturition, Gillespie *et al*. found that mirabegron (β_3_-AR agonist) inhibited only non-voiding activity in a rat model of OAB, whereas tolterodine (antimuscarinic) inhibited non-voiding activity, as well as the amplitude of voiding contractions.^15^

Immunohistochemical staining has identified β_3_-AR not only on detrusor muscle, but also on the afferent terminals of the dorsal root ganglia of L6-S1^16^, suggesting that β_3_-AR agonism might directly affect afferent transmission. Several studies have shown β-AR agonist-mediated disruption of afferent nerve signals. As mentioned, Aizawa *et al*. showed that the β_3_-AR agonist, CL316243, was able to inhibit filling-induced Aδ-fiber signaling and C-fiber mediated hyperactivity caused by PG E_2_.^14^ These results were supported by the study by Kanai *et al*., who found that the β_3_-AR agonist, BRL37344, directly inhibited stretch-induced afferent firing in spinal cord transected mouse bladder sheets.^16^

Investigations of the bladder mucosa have shown that the mechanism of β_3_-AR agonism might involve interaction with the urothelium/suburothelium. The β_3_-AR shows intense immunohistochemical staining in the urothelium and suburothelial interstitial cells.#b^17^b^18^ It has been shown that norepinephrine can stimulate the release of nitric oxide from the urothelium,^19^ but β_3_-AR stimulation might also release other factors. Murakami *et al*. studied the inhibition of carbachol-induced pig detrusor contractions by isoprenaline and concluded that the relaxation responses did not involve the urothelium, and was not caused by NO.^20^ A similar study by Otsuka *et al*. found that the presence of the urothelium caused a right-ward shift of the concentrations–response curve for isoprenaline.^21^ They suggested that this was a result of β-AR mediated release of an inhibitory factor from the urothelium, which inhibited the β-AR agonist-induced relaxation of the detrusor. However, the influence of stimulation of urothelial β_3_-AR on detrusor muscle contraction and on afferent signaling requires further study.

Mirabegron is a β_3_-AR selective agonist that has recently been approved for the treatment of OAB in Japan and the USA, and is under consideration in Europe. Phase IIa, IIb and III drug trials have shown that mirabegron consistently improved the mean number of micturitions in 24 h, and the number of continence episodes in 24 h.#b^22^b^23^b^24^b^25^ The reported adverse events were similar in placebo and mirabegron groups, and included hypertension, headache, gastrointestinal symptoms, dyspepsia and nausea. There was also a small increase in pulse rate in both trials, but no adverse cardiovascular events were reported. These studies clearly establish β_3_-AR agonism as an effective treatment of OAB.

### PDE5 inhibition

LUTS in men include storage symptoms. It is well documented that PDE5 inhibitors improve male LUTS, whether or not these symptoms are associated with erectile dysfunction.^26^ The exact mechanisms by which these beneficial effects are exerted have still not been established,^27^ but this has stimulated research activities with respect to the different signaling pathways controlling the function of the LUT. PDE5 is expressed and has biological activity in all parts of the genitourinary tract, but with respect to its role in LUTS pathophysiology, focus has been on the prostate, bladder and urethra.#b^26^b^28^b^29^ Although PDE5 inhibition *in vitro* can relax the smooth muscle of the LUT, and specifically the outflow region, symptom improvements have not been associated with significantly improved urinary flow rates,^30^ implying that there might be some other mechanism(s) involved. PDE5 inhibitors have several effects on the LUT, and as discussed by, for example, Andersson *et al*.,^27^ they have shown effects not only on smooth muscle, but also on endothelial cell proliferation, nerve activity and tissue perfusion, all factors that might impact LUTS in men. Interestingly, the mucosal effects of PDE5 inhibitors do not seem to have been studied specifically.

Several *in vitro* studies on LUT smooth muscle have highlighted the involvement of the NO/cGMP pathway in the mechanism of action of PDE5 inhibitors. Oger *et al*. investigated the effect of sildenafil on the smooth muscle of the human bladder dome contracted by carbachol and found that sildenafil had a direct relaxant effect.^31^ However, high concentrations of the drug were needed. The relaxant effects were suggested to involve the cGMP pathway, as well as K^+^ channels. As the relaxation remained unaltered in the presence of the NO donor, sodium nitroprusside, the authors questioned the role of NO and the CGMP system in human bladder dome smooth muscle. This site of action might not be predictive of the *in vivo* effects of the PDE5 inhibitors.

In smooth muscle isolated from the periurethral and transition zones of non-diseased human prostates, Ückert *et al*. carried out *in vitro* studies showing that PDE5 inhibitors caused a dose-dependent decrease of the tension induced by norepinephrine.^32^ The relaxant effect, which was associated with increases in cGMP concentrations, varied between 17 and 52%. In isolated human female urethral smooth muscle, the contraction induced by noradrenaline was relaxed in response to sildenafil, vardenafil and tadalafil,^33^ but high concentrations of the drugs were needed. However, nerve-induced relaxations were enhanced at low drug concentrations. In isolated preparations from the human male proximal penile urethra, the relaxation of norepinephrine contracted preparations produced by sildenafil, vardenafil and tadalafil were modest (20–35%), and associated by elevations in cGMP.^34^ The predictive value of these experiments for estimating the effects on human urethral pressure do not seem convincing.

*In vivo* studies have suggested that NO or its downstream signaling could modulate the micturition reflex by reducing the excitability of bladder afferents. Caremel *et al*. evaluated the role of the NO/cGMP signaling pathway on the micturition reflex in a model of bladder hyperactivity associated with C-fiber activation in the rat.^35^ They confirmed previous studies#b^36^b^37^ that compounds inhibiting the NO/cGMP pathway increased bladder overactivity, whereas compounds activating the NO/cGMP pathway inhibited it. Similar studies have found that nitric oxide generation of cGMP might be the key step in the NO/cGMP pathway responsible for PDE5 inhibition of bladder overactivity.#b^38^b^39^

Several lines of evidence further suggest that PDE5 inhibition might affect afferent signaling. PDE5 increases the accumulation of cGMP, which in turn stimulates the activity in PKG. Increased PKG activity might decrease influx through N-type voltage-gated Ca2^+^ channels in afferent nerve terminals, resulting in decreased neuropeptide release.#b^40^b^41^ This would also reduce positive feedback on presynaptic NK1 and NK2 receptors, which in turn might decrease afferent firing.#b^41^b^42^b^43^ Studies have shown direct inhibition of afferent nerve transmission. For example, in a model of bladder hyperactivity involving unanesthetized, decerebrate SCI rats, Behr-Roussel *et al*. found that administration of vardenafil reduced bladder afferent nerve firing, as well as non-voiding contractions.^44^ Minagawa *et al*. found that tadalafil decreased afferent signaling in Aδ and C-fibers in response to both bladder filling and hyperactivity caused by acrolein-induced cystitis with no change in bladder tone.^45^ This decrease in afferent signaling seemed to be related to PDE5 inhibitor-mediated increased activity in the NO/cGMP pathway, as the administration of L-arginine inhibited Aδ and C-fiber firing, whereas the NOS inhibitor, L-NAME, increased afferent activity.^46^ It might be questioned if a direct effect on afferent nerves is the only mechanism by which PDE5 inhibitors influences afferent signaling. Assuming that pelvic ischemia contributes to LUTS, improvement of blood flow to the LUT would be expected to also improve LUT function. In a model of chronic bladder ischemia where vascular occlusion was produced by balloon-induced endothelial injury of the iliac arteries combined with a high cholesterol diet, Nomiya *et al*. found that chronic treatment with tadalafil, even if it did not prevent neo-intimal formation and luminal occlusion, had beneficial effects on bladder function.^47^ It reduced bladder overactivity, decreased indicators of bladder ischemia, normalized changes in NOS activity (decreases of nNOS and eNOS, increase of iNOS) and prevented collagen deposition.

Thus, there might be several mechanisms by which PDE5 inhibitors affect the bladder, all resulting in reduced afferent signaling, which seem to be the final common pathway of relevance for the generation of LUTS.

To date, only tadalafil has been approved for the treatment of LUTS secondary to BPH. RCT have shown significant improvement in urinary symptoms, and the drug is well tolerated.#b^48^b^49^b^50^b^51^b^52^ However, further studies on the long-term effects are required, and whether or not the drug can improve storage symptoms in women remains to be established.

### Botulinum toxin

BoNT, the neurotoxin produced by *Clostridium botulinum*, comprises seven subtypes, of which subtype A (BoNT-A), which has the longest duration of action, is clinically the most relevant. BoNT-A is available in three different commercial forms, which differ in their relative potency: onabotulinum toxin A, abobotulinum toxin A and incobotulinum toxin A. Although there are differences in potency between the forms, there are no reasons to believe that their basic mechanisms of action is different. Most of the information available preclinically and clinically derives from the use of onabotulinum toxin A.

The details of the mechanisms of action of BoNT in the nerve terminal are well-outlined elsewhere.#b^53^b^54^ Briefly, it involves cleavage of the attachment proteins involved with the mechanism of fusion of synaptic vesicles to the cytoplasmatic membrane necessary for neurotransmitter release. Attachment proteins (the SNARE complex) include SNAP 25, synaptobrevin (vesicle associated membrane protein) and syntaxin. BoNT-A cleaves SNAP 25, rendering the SNARE complex inactive.#b^53^b^54^ In striated muscle, paralysis is produced by inhibition of ACh release from cholinergic motor nerve endings.^53^

In the human bladder, SNAP-25 expression has been shown in parasympathetic, sympathetic and sensory fibers.^55^ Blockade of ACh release is believed to play an essential role in the detrusor hypo- or acontractility that follows BoNT-A injection in the bladder. Supporting this view, in normal or SCI animals, BoNT/A treatment decreased the bladder contractions evoked by electrical stimulation of spinal nerves without altering autonomous contractile activity.^56^ However, BoNT-A might also have effects on sensory fibers, as approximately half of the peptidergic sensory fibers express SNAP 25.^55^ It has been well documented that BoNT-A can inhibit release from sensory nerves both in the CNS and peripherally.#b^57^b^58^b^59^b^60^ BoNT-A was found to reduce afferent firing from bladder afferents and antidromic release of neuropeptides.^56^

Although SNAP 25 immunoreactivity has not been detected in urothelial cells,^55^ urothelial function also seems compromised after BoNT-A administration. BoNT-A has been shown to inhibit ATP release from the urothelium in animal models of spinal cord injury.#b^61^b^62^

In addition to its effect on neurotransmitter release, BoNT-A, injected into the bladder wall seems to influence the receptor profile of important neurotransmitters. Apostolidis *et al*. found that the mucosal levels of P2X3 and TRVP1 were decreased 4 weeks after BoNT-A injection, and even more so after 16 weeks.#b^63^ The decrease in the levels of these receptors seemed to correlate with those patients who experienced decreased urgency after the injection. Datta *et al*. found that patients with OAB had decreased levels of muscarinic receptors in the urothelium/suburothelium and that the levels of muscarinic receptors 1 and 3 were normalized after treatment with BoNT-A.#b^64^ Furthermore, they found an inverse association with receptor level and patient-reported symptoms. The relationship between mucosal receptor profile and patient symptoms indicates that this might be an important effect mechanism of BoNT-A.

Available evidence thus suggests that BoNT-A, by action on both the motor part of the myogenic (release of contractant transmitters) and on the mucosal (release of sensory transmitters) activation pathways, decreases the afferent nervous activity generated by the bladder during filling.

Several RCT have documented the clinical effects of BoNT-A in both neurogenic and idiopathic DO,#b^65^ where the drug decreases incontinence episodes, frequency and urgency, and improves quality of life.#b^66^b^67^b^68^ However, successful OAB treatment with BoNT-A does not appear to be related to the existence of DO. No differences in outcomes were found between those with and those without baseline DO.#b^69^b^70^ BoNT-A is also effective in patients with OAB syndrome not responding to antimuscarinic drugs.#b^69^b^70^

The major adverse effects are urinary retention, sometimes requiring clean intermittent catherization, and urinary tract infections. Beneficial effects were shown not to be dose-dependent, whereas side effects might be lessened at lower doses.#b^71^

## Conclusions

OAB is a filling disorder in which abnormal sensations leads to urinary urgency, frequency and incontinence. The afferent signaling pathways that regulate micturition play a central role in the pathogenesis of OAB, and thus represent important targets for therapy. The three drugs discussed, mirabegron, tadalafil and BoNT-A (although acting through different mechanisms), share a central theme – inhibition of afferent signaling from the bladder.

## Abbreviations

ACh: acetylcholine
AR: adrenoceptor
ATP: adenosine triphosphate
BoNT: botulinum toxin
cGMP: cyclic guanosine monophosphate
CNS: central nervous system
DO: detrusor overactivity
eNOS: endothelial nitric oxide synthase
iNOS: inducible nitric oxide synthase
L-NAME: L-NG-nitroarginine methyl ester
LUT: lower urinary tract symptom
LUTS: lower urinary tract symptoms
NOS: nitric oxide synthase
nNOS: neurogenic nitric oxide synthase
OAB: overactive bladder
PDE5: phosphodiesterase type 5
PKG: cGMP activated protein kinase
PG: prostaglandin
RCT: randomized control trials
SCI: spinal cord injury
SNAP 25: synaptosome-associated protein 25 kD
